# Nicotine delivery to users from cigarettes and from different types of e-cigarettes

**DOI:** 10.1007/s00213-016-4512-6

**Published:** 2017-01-09

**Authors:** Peter Hajek, Dunja Przulj, Anna Phillips, Rebecca Anderson, Hayden McRobbie

**Affiliations:** Health and Lifestyle Research Unit, Queen Mary University of London, 2 Stayner’s Road, London, E1 4AH UK

**Keywords:** E-cigarettes, Vaping, Smoking, Nicotine delivery

## Abstract

**Background:**

Delivering nicotine in the way smokers seek is likely to be the key factor in e-cigarette (EC) success in replacing cigarettes. We examined to what degree different types of EC mimic nicotine intake from cigarettes.

**Methods:**

Twelve participants (‘dual users’ of EC and cigarettes) used their own brand cigarette and nine different EC brands. Blood samples were taken at baseline and at 2-min intervals for 10 min and again at 30 min.

**Results:**

Eleven smokers provided usable data. None of the EC matched cigarettes in nicotine delivery (*C*_max_ = 17.9 ng/ml, *T*_max_ = 4 min and AUC_0–>30_ = 315 ng/ml/min). The EC with 48 mg/ml nicotine generated the closest PK profile (*C*_max_ = 13.6 ng/ml, *T*_max_ = 4 min, AUC_0–>30_ = 245 ng/ml/min), followed by a third generation EC using 20 mg/ml nicotine (*C*_max_ = 11.9 ng/ml, *T*_max_ = 6 min, AUC_0–>30_ = 232 ng/ml/min), followed by the tank system using 20 mg/ml nicotine (*C*_max_ = 9.9 ng/ml, *T*_max_ = 6 min, AUC_0–>30_ = 201 ng/ml/min). Cig-a-like PK values were similar, ranging from *C*_max_ 7.5 to 9.7 ng/ml, *T*_max_ 4-6 min, and AUC_0–>30_ 144 to 173 ng/ml/min. Moderate differences in e-liquid nicotine concentrations had little effect on nicotine delivery, e.g. the EC with 24 mg/ml cartridge had the same PK profile as ECs with 16 mg/ml cartridges. Using similar strength e-liquid, the tank EC provided significantly more nicotine than cig-a-like ECs.

**Conclusions:**

EC brands we tested do not deliver nicotine as efficiently as cigarettes, but newer EC products deliver nicotine more efficiently than cig-a-like brands. Moderate variations in nicotine content of e-liquid have little effect on nicotine delivery. Smokers who are finding cig-a-like EC unsatisfactory should be advised to try more advanced systems.

## Introduction

Electronic cigarettes (EC) are a developing technology aiming to provide nicotine without the harmful chemicals produced by tobacco combustion (Etter 2012). EC have the potential to generate a substantial public health benefit if there is a switch from smoking to EC use on a population scale (Public Health England 2015; RCoP 2016). So far, however, only a minority of smokers who try EC progress to the full switch from smoking to vaping (Douptcheva et al. 2013; Kralikova et al. 2013; Farsalinos et al. 2016). This suggests that the currently available EC products do not yet match combustible tobacco closely enough in providing smokers with what they want from their cigarettes. The nicotine delivery profile is likely to play a major role (Caldwell et al. 2012; McRobbie et al. 2010).

The parameters of nicotine delivery that are likely to be important for smokers include the overall nicotine dose and the speed of nicotine absorption (Schroeder and Hoffman 2014). Several studies compared nicotine yields from cigarettes and from EC using puffing machines (Cobb et al. 2010; Farsalinos et al. 2013; Goniewicz et al. 2014; Goniewicz et al. 2013; McAuley et al. 2012; Pellegrino et al. 2012; Trehy et al. 2011; Westenberger 2009). Earlier EC were shown to deliver much less nicotine than cigarettes, but recent devices used at high power setting have improved nicotine delivery (Farsalinos et al. 2016). Machine yields, however, may not correspond with the actual delivery of nicotine into the blood stream of users and the method provides no information about the speed with which nicotine is absorbed. Some studies measured levels of cotinine, a metabolite of nicotine, in vapers, but this is more difficult to interpret because it is not known what proportion of cotinine is derived from nicotine that has been swallowed; and cotinine levels also provide no data on speed of nicotine delivery.

Some data comparing nicotine concentrations in blood samples from smokers and vapers exist as well. In smokers with no experience of EC use, first generation ‘cig-a-like’ EC delivered nicotine very slowly and at low concentrations (Bullen et al. 2010; Vansickel and Eissenberg 2012a; Vansickel et al. 2012b) suggesting buccal rather than pulmonary absorption. There is some evidence that experience with EC improves nicotine intake (Hajek et al. 2014) and so studies with experienced vapers using their EC ad-lib are probably more informative. In a study with experienced vapers who were, however, still prescribed a puffing schedule, nicotine concentrations reached 7 ng/ml 10 min after a 10-puff bout (Dawkins and Corcoran 2014). For comparison, plasma nicotine concentrations from a single cigarette average 15–20 ng/ml (Benowitz et al. 2006). Ad-lib vaping for an hour generated nicotine concentrations of up to 14 ng/ml in one study (Dawkins and Corcoran 2014) and 16 ng/ml in another (Vansickel and Eissenberg 2012a). In smokers with no experience with EC and a fairly intensive puffing schedule prescribed, 5 min after initiation, venous blood nicotine levels achieved with 8, 18 and 36 mg liquid were 9, 13 and 17 ng/ml (Lopez et al. 2015) while in an identical experiment with experienced vapers, the levels were 18, 26 and 36 ng/ml (Ramôa et al. 2015).

Apart from user experience and puffing characteristics, the type of EC is likely to play a major role. More advanced refillable EC products with stronger batteries delivered nicotine more efficiently than a ‘cig-a-like’ EC in one study (Farsalinos et al. 2014). Cigarettes typically reach time of maximal nicotine concentration (*T*_max_) within a few minutes (Digard et al. 2013) while early first-generation EC achieved *T*_max_ after 20 min (Bullen et al. 2010). Experienced vapers using their own EC, mostly tank systems, though again with a prescribed puffing schedule, averaged maximum plasma nicotine concentration (*C*_max_) of only 8 ng/ml, but the *T*_max_ was 5 min (St Helen et al. 2015). Even higher levels were achieved in a recent study where experienced vapers used an advanced vaping device with 24 mg/ml e-liquid (44 ng/ml after 1 h) (Dawkins et al. 2016).

Up to now, little is known about differences in nicotine delivery between individual EC brands. Among nicotine replacement treatments, those that deliver nicotine faster are more likely to be used long-term (Hajek et al. 2007; Sutherland et al. 1992). It is likely that compared to EC with low and slow nicotine delivery, devices with a faster and higher nicotine delivery will appeal to smokers more and will have a better potential to replace cigarettes and assist with smoking cessation. Other EC characteristics such as taste, ease of use, puff resistance, vapour volume and handling characteristics (and of course cost, as well as product marketing) are likely to be important too, but it can be expected that the nicotine delivery profile will be paramount. One sign of this is that nicotine containing EC dominate the market with nicotine-free models hardly used despite being otherwise equivalent and widely available (Etter 2012). Nicotine delivery, however, may need to fit into a relatively narrow range at both ends. Very high nicotine concentration liquids are also rarely used.

EC technology is evolving and market forces are likely to steer product development to features that appeal to smokers and increase the rate of adoption, but the process could be slow. Data are needed that monitor pharmacokinetic (PK) characteristics of different EC brands to provide information that could guide smokers faced with the wide range of different EC products, inform the choice of EC brands for studies of the potential of EC in smoking cessation, and guide further product development.

In the first study of this type, we tested PK profiles of eight common EC brands, together with conventional cigarettes and with vapers’ own devices. We also adjusted the common methodology in this field to our particular aim. Most studies assessing nicotine intake from alternative nicotine delivery products take the first post-baseline sample 5 min after the initial nicotine intake. We started 2 min after the first puff and followed the changes in nicotine absorption in 2-min intervals to allow for more accurate comparison with smoking and to assess whether speed of nicotine absorption suggest a buccal or a much faster pulmonary route. We also asked experienced vapers to use each device after overnight abstinence ad lib. EC studies up to now have typically used prescribed puffing schedules that do not allow for individual adjustments smokers make when using different products and may not correspond to ‘real-life’ levels of nicotine that vapers obtain from vaping.

## Methods

### Aim and design

A crossover study to establish PK profiles of eight popular EC products and participants’ own EC and to compare them with the PK profile obtained from their own cigarettes; and to assess effects on PK profiles of different e-liquid strengths and different types of EC models.

### Participants

Twelve healthy smokers who were currently smoking and vaping (‘dual users’) and who were willing to test a series of EC products were recruited via UK on-line forums of EC users and by word of mouth.

### Study procedures and setting

Participants were pre-screened over the telephone and attended the laboratory after overnight abstinence from both smoking and vaping.

At the first session, participants smoked a cigarette of their usual brand that they brought with them. At the next session, own-brand EC was tested, followed by eight EC brands, always tested in the same order. Sessions were scheduled with at least 3-day ‘wash out’ periods between them.

The sessions took place between 7.30 and 9.30 am, depending on the participants’ availability, and took about 60 min.

Participants received £60 at the end of each session.

At each session, an intravenous line for blood sampling was placed in the forearm and the baseline blood sample was taken, after which participants were asked to smoke/vape ad-lib for 5 min. Further blood samples were taken at 2, 4, 6, 8, 10 and 30 min after starting smoking/vaping.

### Measures

Demographic and smoking history data were collected at baseline. Subjective product ratings were collected at the end of each session. (This report focuses on nicotine delivery from different types of EC compared to nicotine delivery from cigarettes. Other product characteristics and user ratings from a larger sample that included participants who did not smoke anymore and so did not provide data on nicotine delivery from their cigarette will be covered separately.)

Blood samples were analysed at ABS Laboratories Ltd., BioPark, Broadwater Road, Welwyn Garden City, Hertfordshire, UK. During the 5 min of ad-lib smoking and vaping, investigators kept count of the number of puffs taken.

### Study products

We chose a range of popular first generation products, including all those marketed by major tobacco companies, and examples of the second and third generation EC. The following six first generation (cig-a-like) products were tested: Gamucci (16 mg/ml nicotine, ‘original taste’ (tobacco)), Blu (18 mg/ml nicotine, ‘classic tobacco’), Vype (16.8 mg/ml, ‘classic tobacco flavour’, regular), E-lites (24 mg/ml nicotine, ‘original’ (tobacco)), Puritane (20 mg/ml, ‘original’ (tobacco)) and Vuse (4.8% nicotine (i.e. 48 mg/ml), ‘original’ (tobacco)). The cig-a-like products included the five EC marketed by the tobacco industry (Blu—Imperial Tobacco, Vype—British American Tobacco, Puritane—Imperial Tobacco, E-lites—Japan Tobacco and Vuse—RJ Reynolds), and one product produced by an independent manufacturer and popular in the UK (Gamucci). Using E-lites also allowed testing of a product available with cartridges that have a higher nicotine concentration (24 mg/ml).

We also tested one second generation refillable ‘tank’ product, a popular mid-range KangerTech EVOD, and one third generation product (a tank product which allows variable power setting), Innokin iTaste MVP 2 with an ‘iClear’ atomizer (2.1Ohm), set to 11 W. Both of these refillable products were used with the same 20 mg/ml nicotine e-liquid (Vermillion River ‘classic blend’ (tobacco)).

For products marketed at different strengths, nicotine concentrations were selected to be as close as possible to 20 mg/ml. Across products tested, the e-liquids contained 16–20 mg/ml of nicotine, with two exceptions. We used E-lites with 24 mg/ml of nicotine to assess a cig-a-like product with a higher nicotine content; and Vuse was only available with strength marked as 4.8%, translating into 48 mg/ml. Table 1 provides product details.

**Table 1:** Summary of EC products tested

Brand and manufacturer	Nicotine content per ml as per packaging	Starter pack and cartridge or e-liquid price
Gamucci (Gamucci XL Distributors Inc.)	16 mg	£9.99/ £2.33 per cartridge
Blu (Imperial Tobacco)	18 mg	£14.99/ £2 per cartridge
Vype (British American Tobacco)	16.8 mg	£14.99/ £2 per cartridge
E-lites Original Instant Use (Japan Tobacco)	24 mg	£6.49/ £3.49 per cartridge
Puritane (Imperial Tobacco)	20 mg	£22.99/ £4.50 per cartridge
Vuse (RJ Reynolds)	48 mg	£6.25/ £1.90 per cartridge
KangerTech EVOD (Kanger Technology)	20 mg	£32/ £4.99 per 10 ml e-liquid
Innokin itaste MVP 2, 4.8v (Innokin Technology)	20 mg	£34/ £4.99 per 10 ml e-liquid

Regarding the own brand EC, participants used the following first generation products: Vapestick disposable (18 mg/ml, original), 10 motives (16 mg/ml menthol and 2 × 16 mg/ml, regular) and Vapourlites (11 mg/ml, cherry). The following second generation products were used: KangerTech EVOD (12 mg/ml, menthol - Dekang), Aspire CF G Power battery with BDC cartomiser (9 mg/ml, American tobacco – FLAVaah!), iBaccy (24 mg/ml, chocolate - VIP). The following third generation products were used: E-lectron-S (18 mg/ml, cherry -Totally Wicked), Eleaf iStick subtank (6 mg/ml, fruit punch – Mystic Juice), Aspire nautilus (12 mg/ml sweet prudence - Halcyon Haze), Torchwood battery with atomic atomiser (12 mg/ml vanilla custard – own mix).

### Statistical analyses

PK parameters were calculated using PKSolver add-in for Excel (V2.0 (Zhang et al. 2010)). The following PK parameters were calculated: (1) time at which the highest nicotine concentration occurred in plasma (*T*_max_); (2) the highest drug concentration observed in plasma (*C*_max_); (3) estimated area under the plasma nicotine curve concentration from time 0 to 30 min (AUC_0–>30_). All measures were corrected for baseline values. *C*_max_, *T*_max_ and AUC_0–>30_ were estimated using a non-compartmental model and trapezoidal rule. Differences between products were analysed using paired-sample *t* test. Analyses were performed with SPSS v.22.

## Results

One participant dropped out within the first 5 weeks. The analyses below include the remaining 11 participants. Table 2 provides baseline characteristics of the sample.

**Table 2:** Baseline characteristics (*N* = 11)

Age, mean (SD)	34.1 (12.0)
Male (%)	91
Higher education (%)	73
Cigarettes smoked per day before starting EC use, mean (SD)	12.4 (8.4)
Fagerstrom Test for Nicotine Dependence (FTND) before EC use, mean (SD)	3.4 (2.5)
EC cartridges used per day, mean (SD)^a^	1.5 (1.5)
Millilitres of e-liquid used per day, mean (SD)^b^	3.3 (1.1)
No. months using EC daily, mean (SD)	15.7 (16.2)
Days EC used in last week, mean (SD)	7 (0)
Cigarettes smoked per day currently, mean (SD)^c^	1.6 (2.0)

^a^ *N* = 5

^b^ *N* = 6

^c^Calculated by dividing the answer to the question about cigarettes smoked per week by 7

**Fig. 1:**
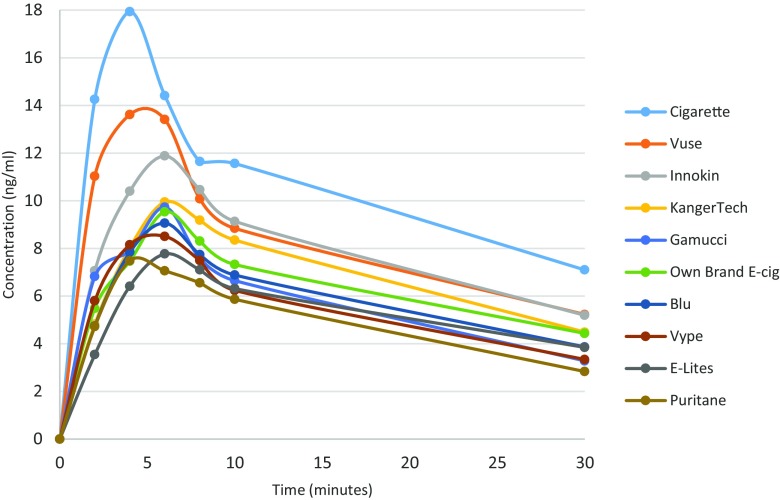
Pharmacokinetic profiles of own brand cigarette and each electronic cigarette product

PK profiles achieved with individual EC products and with own brand cigarettes are shown in Fig. 1.

Table 3 provides PK parameters for each product. None of the products matched cigarettes in terms of nicotine delivery despite the fact that the participants took significantly more puffs from most of them.

**Table 3:** Nicotine delivery and number of puffs from own brand cigarette, own brand EC and eight EC products

Product	Mean no. of puffs (SD)	Mean *C*_max_ (SD)	Mean *T*_max_ (range)	Mean AUC_0–>30_ (SD)
Cigarette	14 (4.5)	17.9 (16.0)	4 (2–30)	314.6 (155.1.)
Vuse	19 (5.1)*	13.6 (9.7)	4 (2–10)	244.9 (116.1)
Innokin	17 (5.2)	11.9 (7.0)	6 (4–30)	232.1 (112.5)
KangerTech EVOD	18 (5.9)	9.9 (6.6)	6 (6–30)	200.6 (120.1.)
Gamucci	18 (5.4)*	9.7 (4.4)	6 (2–10)	169.9* (74.9)
Own Brand EC	18 (5.3)*	9.5 (6.1)	6 (2–30)	186.4* (96.6)
Blu	20 (6.4)*	9.1 (6.8)	6 4–30	173.1* (112.4)
Vype	22 (7.1)*	8.5 (5.7)	6 4–30)	161.0* (98.6)
E-lites	17 (5.1)	7.8 (4.6)	6 (4–30)	157.6* (69.9)
Puritane	22 (11.4)*	7.5 (5.0)	4 (4–10)	144.4* (67.2)

*T*_max_ and AUC values that were used to compare EC products statistically used means across individuals while values shown in Fig. 1 and Table 3 estimated by PK Solver used means across time points

*Significant difference compared to cigarette (*P* ≤ 0.05)

Among the eight EC products tested, Vuse which used an exceptionally high nicotine concentration e-liquid (48 ng/ml) generated the most efficient nicotine absorption, followed by the third generation EC device Innokin using 20 mg/ml nicotine e-liquid, followed by the second generation EC device Kanger using the same e-liquid.

Small differences in e-liquid nicotine concentrations in cig-a-like brands had no bearing on nicotine delivery, e.g. E-lites using 24 mg/ml cartridges had a virtually identical PK profile to Gamucci and Vype with 16 mg/ml nicotine concentrations.

Participants took a greater number puffs when using EC compared to cigarettes; the differences were significant for all EC products except the two refillables (KangerTech and Innokin) and the 24 ml/mg E-lites.

Table 4 shows PK parameters of the five cig-a-like products other than Vuse (Blu, Puritane, Vype, Gamucci and E-lites and the two refillable products (KangerTech and Innokin). Refillable EC produced significantly higher *C*_max_ and AUC_0–>30_.

**Table 4:** Nicotine delivery from cig-a-likes and refillable EC products (*N* = 11)

Measure	EC type		Difference
	Cig-a-like EC	Re-fillable EC	
*C*_max_ (SD)	8.5 (4.6)	11.7 (6.5)	*P* = 0.044
*T*_max_ (range)	6 (2–30)	6 (4–30)	ns
AUC_0–>30_ (SD)	158.0 (70.0)	235.2 (80.4)	*P* = 0.001
No. puffs (SD)	20.0 (6.3)	17.5 (5.3)	ns

## Discussion

The main findings of this study provide a new insight into EC products when used by experienced vapers ad-lib. EC brands we tested deliver less nicotine than cigarettes and do it more slowly despite users taking significantly more puffs than when using cigarettes. The maximum nicotine concentrations of under 10 ng/ml achieved only after some 6 min suggest that nicotine may have been delivered largely via buccal rather than pulmonary absorption. However, Vuse, an EC with a very high nicotine content, and the third generation EC we tested at a high power setting, are beginning to catch up with cigarettes. With the exception of Vuse, different cig-a-like products produced by different manufacturers may differ in appearance and various other characteristics, but in terms of nicotine delivery, they are practically identical. Refillable EC brands using similar strength of e-liquid delivered nicotine significantly more efficiently.

The main limitation of the study is the small sample size. Individual participants differed in their nicotine intake from different products and the results may have been influenced by ‘outlier’ values. Another limitation is that although the participants were experienced vapers, they had no prior experience with the products tested. This means that they may have not used the tested EC in an optimal way or in a way they would use them if they were familiar with them. Superficial characteristics such as product flavour or draw resistance may have influenced product use and therefore nicotine intake. The results could have been different after a period of familiarisation with each product. We also did not verify nicotine content of EC we used, but our previous study suggested that the labelling of popular EC products is reasonably precise (Goniewicz et al. 2014). Finally, because obtaining regulatory approvals for any study with human subjects takes many months and trial completion and publication takes time as well, publications in this area of fast technological developments will never report on the newest EC models. The most recent EC products are likely to provide better nicotine delivery than the products we tested.

Within these limitations, the key results seem fairly robust. EC products that we tested do not yet match cigarettes in nicotine delivery profile. The developmental trajectory, however, seems to be moving to EC products that deliver nicotine faster. Second and third generation EC deliver nicotine more effectively than ‘cig-a-likes’. One EC that was an outlier is Vuse, a ‘hybrid’ product that uses a large cartridge and a much stronger e-liquid.

Smokers typically start with cig-a-like EC but those who switch completely to EC are more likely to use tank systems (ASH 2015). Our results suggest that this is in part at least linked to their better nicotine delivery. It is of interest to note that five of the study participants used cig-a-like products and almost all used weak or ‘mild’ e-liquids. This may be because they did not discover better products yet and that could also be the reason that they remained dual users rather than switching to vaping altogether, but the observation could also reflect the fact that the participants were on average relatively light smokers before they started to vape. While EC with better nicotine delivery are likely to be more helpful for heavy smokers, it is important to note that not all smokers seek high nicotine concentrations. It is also highly likely that other chemicals in tobacco smoke apart from nicotine contribute to the attractiveness of smoking. Unlike cigarettes that satisfy smokers and encourage non-smokers to start regular use, nicotine on its own, e.g. in NRT products and, up to now, in EC, has limited appeal to smokers (Kralikova et al. 2013) and no appeal to non-smokers (ASH 2015; Brown et al. 2014; Gerlach et al. 2008).

There are large differences between individual vapers in their requirement for nicotine and in what they want from EC use. Participants in our study differed widely in nicotine concentrations obtained from their own EC. Availability of a range of different EC products is likely to increase the likelihood that smokers find an EC that fits their needs.

In terms of practical implications of our findings, heavy smokers who are finding cig-a-like EC unsatisfactory should be advised to try tank products or a cartridge EC with a high nicotine concentration. Smoking cessation trials seeking to evaluate EC with the best treatment potential should offer tank EC with a choice of liquid strengths. Manufacturers should be encouraged to continue developing products that provide smokers with what they seek from their cigarettes while maintaining maximum product safety.

In conclusion, EC products that we tested do not deliver nicotine as efficiently as cigarettes, but refillable EC deliver nicotine more efficiently than cig-a-like products. Moderate variations in nicotine content of e-liquid have little effect on nicotine delivery. Smokers who are finding cig-a-like EC unsatisfactory should be advised to try more advanced systems, as should studies seeking to evaluate EC treatment potential.
